# AAV Assembled Capsids Are Produced in Cells Blocked From Cell Cycle Progression

**DOI:** 10.1002/bit.70111

**Published:** 2025-11-18

**Authors:** Alaka Mullick, Audrey Morasse, Melanie Leclerc, Ziying Liu, Qing Yan Liu, Sonia Leclerc, Milica Momcilovic, Annie Viau, Amine A. Kamen

**Affiliations:** ^1^ Human Health Therapeutics National Research Council of Canada Montreal Quebec Canada; ^2^ Digital Technologies Research Centre National Research Council of Canada Ottawa Ontario Canada; ^3^ Department of Bioengineering McGill University Montreal Quebec Canada; ^4^ Present Address: Gene Therapy Technical Research and Development F. Hoffmann‐La Roche AG Basel Switzerland

**Keywords:** AAV, adeno‐associated virus, cell cycle, E1A, RNA‐seq

## Abstract

Adeno‐associated virus (AAV) is a promising delivery system for gene therapy. However, current manufacturing of AAV suffers from very low yields compared to other biotherapeutics. The AAV dose per patient ranges between 10^11^and 10^15^ viral genomes (vg), requiring an average of 10 to 30 L production/dose. As a consequence, production costs are prohibitive for most indications. Our recent studies revealed that only 10% of the HEK293 cells that have received the AAV encoding DNA produce assembled AAV capsids. This observation prompts the question: Why would cells that have been successfully transfected, be unable to produce AAV. To answer this question, we undertook a detailed study to characterize the two sub‐populations from the same transfection, the cells that were making assembled capsids and those that were not. We found that the two populations had distinct cell cycle profiles, with a block in cell cycle progression characterizing the producer population. RNA‐seq analysis of the two populations reveals differences in the molecular pathways impacted and provides a basis for making changes to improve productivity.

## 1 Introduction

Adeno associated virus (AAV), a member of the *Dependoparvoviridae* genus of the *Parvoviridae* family of viruses is an attractive candidate for in vivo gene therapy, given its low immunogenicity, wide tropism and the possibility of achieving long‐term therapeutic gene expression. Indeed, it has been tested in numerous clinical trials with clear benefits to patients for certain indications (Wang et al. 2024). Most of the earlier trials involving localized administration to the eye required about 10^11^ vg/patient (Bordet and Behar‐Cohen 2019) and therefore did not pose a major manufacturing challenge. However, systemic administration in adult patients requiring 10^16^ vg/patient (Wilson and Flotte 2020), is significantly more challenging and calls for a quantum leap in manufacturing yields.

Present methods involving triple transfection in adherent or suspension human embryonic kidney (HEK 293) cells are limited not only by viral titers, but also the proportion of capsids that carry the viral genome. Depending on the system, the percentage of full capsids ranges from 3% to 30% of the total vector production, requiring additional purification steps to remove empty capsids (Qu et al. 2007). That this is not an inherent problem of AAV is evident from the fact that the wild‐type (wt) AAV can produce 10^5^–10^6^ copies per cell or about 10 times more than transient transfection (Sha et al. 2021), the majority with encapsidated genomes (Zeltner et al. 2010). These observations suggest that it is the delivery of the viral components to the cells, that is inefficient in the transfection process. For instance, in contrast to the wt AAV, the viral protein coding sequences and the vector encapsidation sequence are separated and delivered on two different plasmids, with the purpose of avoiding production of replication‐competent AAV. In addition, the challenges of delivering the necessary genetic elements to the nucleus with appropriate kinetics, have been described by Sha and colleagues (Sha et al. 2021) and they include among the possible limitations, loss of DNA due to lysosomal and then cytoplasmic degradation and transport across the nuclear pore. As a result, a very small fraction of the transfected DNA makes it to the nucleus. Nguyen *et a*l (Nguyen et al. 2021) undertook a detailed study of the kinetic challenges of AAV production by triple transfection and their analysis highlights the lack of synchronization between capsid synthesis and DNA replication resulting in a high fraction of empty capsids.

We have identified another important limitation in that only 10% of the transfected population produces significant quantities of assembled capsids 48 h posttransfection (Dash et al. 2022). This was surprising, since these cells have received DNA in their nuclei. We, therefore undertook a study to understand why these cells did not produce functional AAV vectors, in spite of receiving all essential DNA coding sequences.

## 2 Materials and Methods

### 2.1 Plasmids and Cloning

For the production of rAAV‐GFP, plasmids pAAV‐GFP, pRC2, and pHelper were purchased from Cell Biolabs (San Diego, CA). For a mock transfection, plasmid backbones with no viral coding sequences were transfected in a parallel transfection (pCMV‐GFP). For this transfection, pCMV‐GFP was generated by sequentially removing the ITRs from pAAV‐GFP. Filler plasmids for pCMV‐GFP transfection were generated from pRC2 and pHelper to contain only the sequences of the pRC2 and pHelper backbones (Figure S1). Constructed plasmids were submitted to Sanger sequencing for sequence validation.

### 2.2 Transient Transfections

HEK293 cells adapted to serum‐free suspension culture (clone 293SF‐3F6) cells are described elsewhere (Durocher et al. 2007). Suspension cultures were maintained in 125 mL baffled shake flasks with vented caps (Corning, Oneonta, NY) in Hycell TransFx‐H medium (Hyclone, South Logan, UT) supplemented with 4 mM l‐glutamine (Hyclone) and 0.1% Kolliphor (Sigma‐Aldrich, St Louis, MO) at 37°C, 5% CO_2,_ with agitation set at 120 rpm.

All transfections in suspension culture were performed at a cell density of 8 × 10^5^ cells/mL using a 1:2 mix of plasmid DNA and 25 kDa linear polyethylenimine (Polysciences, Warrington, PA). The final plasmid DNA concentration was 1 μg/mL in all instances. For vector production, plasmids pAAV‐CMV‐GFP, pRC2, and pHelper were mixed at a 1.4:1:1 molar ratio. To mimic the transfection setup for AAV assembly, plasmids pCMV‐GFP, pFiller‐RC2, and pFiller‐Helper were mixed at the same molar ratio for pCMV‐GFP transfection. Cells were maintained for 24 h post transfection, unless specified otherwise.

### 2.3 Intracellular Labeling and Cell Sorting

A20R antibody (Catalogue #. 610298, Progen, Heidelberg, Germany) conjugated with Alexa Fluor 568 dye (Invitrogen, Waltham, MA) was utilized to stain for assembled AAV2 capsids (A20R‐AF568). pCMV‐GFP‐transfected and un‐transfected cells underwent the same staining treatment. At the indicated time posttransfection, fixation, permeabilization and staining was based on a method described by Phan and his colleagues (Phan et al. 2021). Flow cytometry data was acquired using BD FACSDiva software version 8.0.3 and analyzed with FlowJo software version 10.10.0 (BD Biosciences). Sorted cells were collected in PBS containing 1% BSA, which was supplemented with 40 U/mL RNase inhibitor (NEB, Ipswich, MA) in case of RNA extraction.

### 2.4 RNA Extraction From Cell Populations Sorted Using Intracellular Immunolabeling

RNA extraction was based on a method described by Phan and his colleagues (Phan et al. 2021). The cells were washed twice before being resuspended in a 1:1 mixture of PBS and Drop‐seq lysis buffer (Ficoll PM‐400 6%, Sarkosyl 0.2%, EDTA 10 mM, Tris pH 7.5 200 mM, DTT 50 mM), supplemented with Proteinase K (Qiagen). Samples were heated at 56°C for an hour to reverse cross‐linking, followed by an incubation for 10 min at RT and an incubation on ice for at least 5 min. The lysates were then combined with 350 uL of RLT plus buffer from RNeasy kit and processed according to the manufacturer's protocol. The RNA samples thus obtained were quantified and assessed for integrity using the 4200 TapeStation system (Agilent, Santa Clara, CA).

### 2.5 RNA‐seq Analysis

RNA‐seq Libraries were generated by using the NEBNext rRNA Depletion kit v2(Human/Mouse/Rat) followed by Section 2 of the NEBNext® Ultra™ II Directional RNA Library Prep Kit for Illumina (NEB). RNA‐Seq libraries were quantified by qPCR according to the Illumina Sequencing Library qPCR Quantification Guide and the quality of the libraries was evaluated on Agilent Bioanalyzer 2100 using the Agilent DNA‐1000 chip. The RNA‐seq library sequencing was performed using Illumina Next‐Seq. 500. RNA‐seq data were processed by trimming the adaptor sequences, filtering low‐quality reads (Phred Score < = 20) and eliminating short reads (length < = 20 bps) using software package FASTX‐toolkit [http://hannonlab.cshl.edu/fastx_toolkit/]. STAR (v2.7.11a) (Dobin et al. 2013) was used for alignment of the reads to the reference genome and AAV sequence to generate gene‐level read counts (Liu et al. 2023). Human (*Homo sapiens*) reference genome (GRCh38.p14, release 46) (Frankish et al. 2023) and corresponding annotation were used as reference for RNA‐seq data alignment process. DESeq. 2 (Love et al. 2014) was used for data normalization and differentially expressed gene identification for AAV‐producer, non‐producer and pCMV‐GFP versus 239SF, respectively. Differentially expressed genes (DEGs) were defined as *p*‐value less than 0.01 and twofold of changes in ratio (abs(log2 fold‐change) ≥ 1).

### 2.6 RT‐ddPCR and q‐PCR

Two‐step RT‐ddPCR or q‐PCR was employed to quantify AAV, Ad helper transcripts and cellular transcripts. Total RNA was converted to cDNA using the QuantiTect Reverse Transcription Kit (Qiagen, Chatsworth, CA) as per the manufacturer's instructions. Targeted cDNA species were separately quantified with the QX200 ddPCR system (Bio‐Rad Laboratories, Hercules, CA). All assays were EvaGreen‐based and raw data was processed in QX Manager software (Bio‐Rad Laboratories, Hercules, CA). **Quantitative real‐time PCR (qPCR)** was performed using PowerTrack™ SYBR™ Green Master Mix (Applied Biosystems) on a QuantStudio 5 Real‐Time PCR Detection System (Applied Biosystems). Primer sequences and annealing temperatures can be found in Supplementary Table 1.

### 2.7 Western Blot

At the indicated time post‐transfection, cells were stained with antibody A20R‐AF568 and FACS sorted as described above without using the RNase inhibitor. Total protein was extracted from stained and fresh cells following the protocol reported by Sadick and colleagues (Sadick et al. 2016). Membranes were blocked for 1 h with PBS 1% fat‐free milk powder and probed overnight with single antibody as follows: mouse anti‐AAV2 VP monoclonal antibody (Catalogue # 03‐61069, American Research Products, Waltham, MA) was used at 1:2000 dilution to detect VP1, VP2, and VP3; mouse anti‐AAV2 Rep monoclonal antibody from clone 303.9 (Catalogue # 03‐61058, American Research Products, Waltham, MA) was used at 1:100 dilution to detect all four Rep proteins; rabbit anti‐E4orf6 polyclonal antibody was a kind gifted from Dr. Jose Teodoro (McGill University) and was used at 1:2500 dilution to detect E4orf6 protein; mouse anti‐DBP (E2A) monoclonal antibody from clone B6 (Reich et al. 1983) was a kind gift from Dr. Arnold J. Levine (Princeton University) and was used at 1:1000 dilution to detect E2A protein. On the following day, the membranes were washed three times in 1X PBS with 0.1% Tween 20 for 5 min and incubated with a secondary antibody diluted in blocking solution for 1 h at room temperature. Donkey anti‐mouse antibody conjugated with horseradish peroxidase (HRP) (Catalogue # 715‐036‐151, Jackson Immunoresearch Laboratories, West Grove, PA) served as the secondary antibody at 1:10000 dilution except for the E4orf6 target in which case the HRP‐conjugated donkey anti‐rabbit antibody (Catalogue # 711‐036‐152, Jackson Immunoresearch Laboratories, West Grove, PA) was used at 1:10000 dilution. The chemiluminescence signal was detected using the Pierce ^TM^ ECL western blot analysis substrate (Thermo Fisher Sciences catalogue # 32209) by the Azure 600 imaging system (Azure Biosystems, Dublin, CA).

### 2.8 DAPI Staining for Cell Cycle Analysis

DAPI staining was used to evaluate the cell cycle distribution of AAV‐producer and non‐producer sub‐populations. The cells were stained with antibody A20R‐AF568 as described in the previous section and then incubated in PBS containing 1% FBS (Thermo Fisher Scientific, catalogue # SH30071.03 (Cytiva)) 56°C heat‐inactivated in‐house) and 10 μg/mL DAPI stain (Abcam, Cambridge, UK) for 30 min at room temperature. Stained cells were assessed for DAPI signal on the FACS LSRFortessa flow cytometer (Becton Dickinson, San Jose, CA) using a 405 nm laser and a 450/50 bandpass filter. Flow cytometry data was acquired using BD FACSDiva software version 8.0.3 and analyzed with FlowJo software version 10.10.0 (BD Biosciences).

## 3 Results and Discussion

As a first step, we wanted to examine the expression of elements required for AAV production. Transient transfection, which remains the most widely used method for producing AAV vectors, typically entails a cotransfection of 3 plasmids in HEK293 cells, grown in suspension (293SF) or in adherent culture (293 A and 293 T). Successful production of AAV requires a complex interaction between AAV, adenoviral helper proteins and cellular machinery (Meier et al. 2020). Although adenoviral helper genes E2A (DBP), E4orf6 and the VA RNA are delivered by transfection along with the AAV Rep and Cap genes, the HEK293 cells constitutively express the remaining helper genes, adenoviral E1A and E1B given that a fragment of the adenoviral genome is inserted in the HEK293 genome (Louis et al. 1997).

### 3.1 Expression of AAV and Adenoviral Helper Genes

Given that 50%–60% of the transfected pool receives DNA, as judged by GFP expression, and only a small fraction (10%) of these GFP‐expressing cells, produce assembled capsids, 48 h posttransfection (Dash et al. 2022), we wanted to separate AAV‐producing and nonproducing cells from the same GFP^+^ population, and identify differences in gene expression profiles linked to this observation. Since early events are determining factors for AAV biogenesis, productions were harvested 24 h posttransfection for this study.

**Figure 1:**
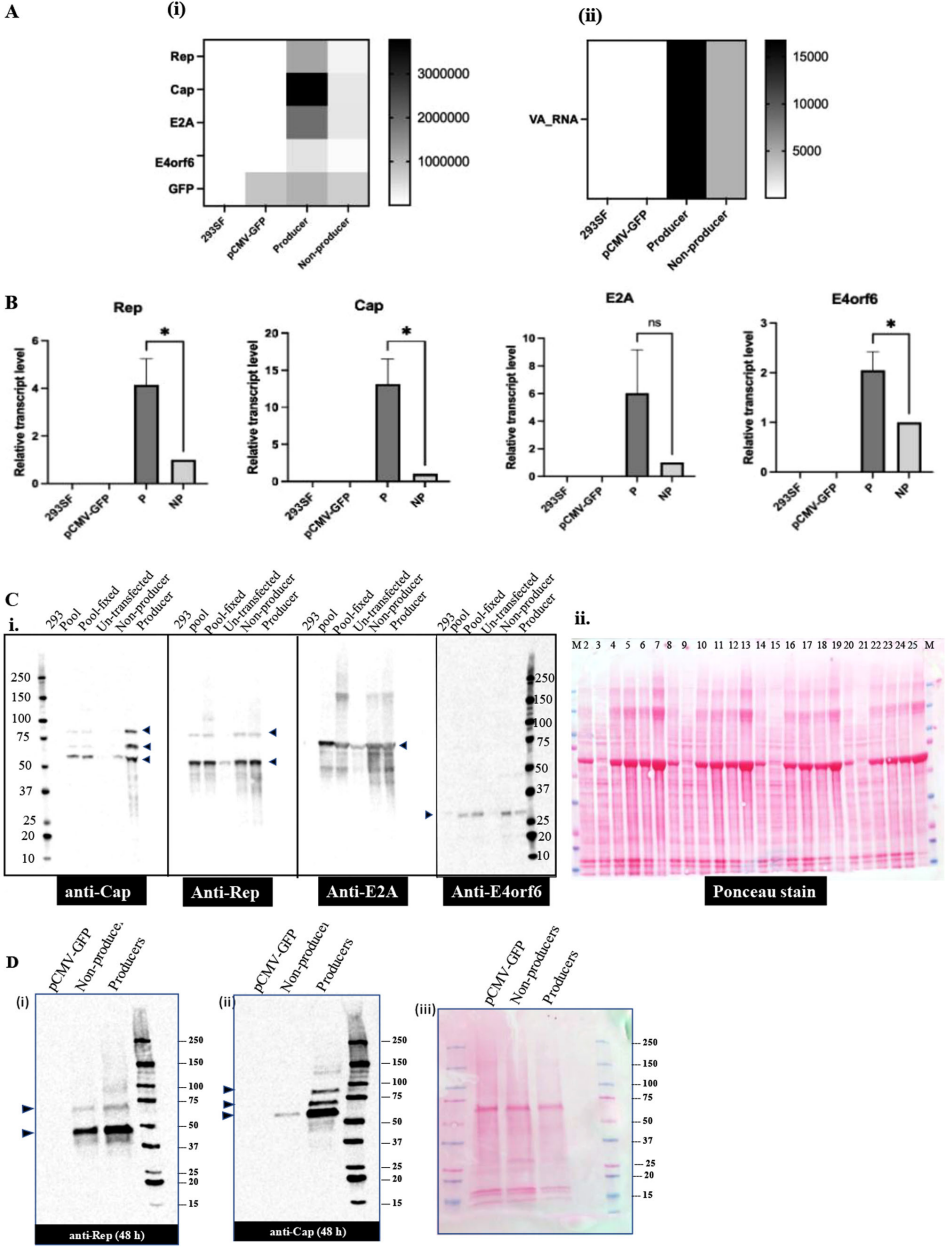
Expression of transfected genes. A. RNA‐seq analysis of transfected genes. Cells were harvested from a triple transfection for AAV production. After fixation, permeabilization and staining as described in Materials and Methods, the populations GFP^+^AF^−^ (non‐producer) and GFP^+^AF^+^ (producer) were sorted based on GFP expression and A20R‐AF568 labeling. Similarly, cells were harvested, stained and sorted to isolate the GFP^+^ population in the pCMV‐GFP transfection. 293SF cells were also fixed, stained and permeabilized. Total RNA was extracted, sequenced and analyzed as described in Materials and Methods. The figure shows a heat map representing average normalized transcript levels from three independent experiments for (i) Rep, Cap, E2A, E4orf6 and GFP and (ii) VA‐RNA. B. RT‐ddPCR analysis of transfected genes RNA was subjected to RT‐ddPCR analysis as described in Materials and Methods. The data was normalized to the GFP^+^AF^−^ sample within the experiment. The figure represents the average +/− standard deviation (s.d.) of normalized transcript levels from three independent experiments. Graph pad prism was used to plot data and perform *t*‐tests to determine statistical significance. *p < 0.05. C. Western blot analysis of transfected genes 293SF were transfected to produce AAV as described in the Materials and Methods section. Cells were harvested 24 h post‐ transfection. 1 × 10^6^ cells from the transfected pool were retained and the rest were fixed, permeabilized and stained as described in Materials and Methods, following which, the populations GFP^‐^AF^−^ (un‐transfected), GFP^+^AF^−^ (non‐producer) and GFP^+^AF^+^(producer) were collected using FACS. 293SF cells were also fixed, stained and permeabilized. Total protein was extracted and subjected to western blot analysis (i) to detect Cap, Rep E2Aand E4orf6. Panel (ii) represents the image of the membrane stained with Ponceau to visualize the proteins loaded. Comparison of the transfected pool with and without fixation shows that fixation did not impact the results. Arrows represent the positions of (i) Rep 68 and 52 (ii) VP1, VP2 and VP3 (iii) E2A and (iv) E4orf6 D. Western blot analysis of Rep and Cap Cells were harvested 48 h posttransfection and were fixed, permeabilized and stained as described in Materials and Methods, following which, the populations GFP^+^AF^−^ and GFP^+^AF^+^ were collected using FACS. Similarly, cells were harvested, stained and sorted to isolate the GFP^+^ population in a pCMV‐GFP transfection Total protein was extracted and subjected to western blot analysis to detect (i) Rep (ii) Cap. Panel (iii) represents the image of the membrane stained with Ponceau to visualize the proteins loaded. The figure shows representative images of 2 experiments. Arrows represent the positions of (i) Rep 68 and 52 (ii) VP1, VP2 and VP3.

293SF‐3F6 (293SF) cells were transfected either with plasmids for AAV production (pAAV‐CMV‐GFP, pRC2 and pHelper) or with plasmids lacking viral coding sequences as a control pCMV‐GFP transfection (pCMV‐GFP, pFiller‐RC2 and pFiller‐Helper) as described in Material and Methods section and Figure S1). Cells from both transfections were harvested 24 h posttransfection, fixed, permeabilized and labeled with an antibody that recognizes assembled AAV capsids (A20R), conjugated with a fluorescent dye (AF568), as described in the Material and Methods section. Cells were subjected to fluorescence‐based cell sorting and the following populations were isolated from the AAV‐producing transfection: cells expressing GFP and recognized by the A20R‐AF568 antibody, GFP^+^AF^+^, defined as AAV‐producers and cells expressing GFP, but not recognized by the A20R‐AF568 antibody, GFP^+^AF^−^, referred to as non‐producers. The GFP^+^ population was similarly isolated from the pCMV‐GFP transfection. Total RNA was extracted and analyzed by RNA‐seq as described in Materials and Methods. Figure 1A shows normalized transcript levels of the transfected genes. Producer cells have higher transcript levels for all 5 transfected genes (4.1x, 7.7x, 3.9x, 2.8x and 2.7x for Rep, Cap, E2A, E4orf6 and VA RNA respectively), although the differences for all 5 viral transcripts is higher than that for GFP (1.2x). These results were confirmed by RT‐ddPCR in three independent experiments (Figure 1B). To determine whether protein levels followed a similar pattern, whole cell protein extracts from two independent experiments were subjected to Western blot analysis, and as shown in Figure 1C, protein levels of Rep, E4orf6 and E2A were similar in both populations. However, the expression levels of capsid proteins, VP1, VP2 and VP3 were significantly lower in the non‐producer population. These differences persisted 48 h post transfection (Figure 1D).

### 3.2 Adenoviral E1A and E1B

**Figure 2:**
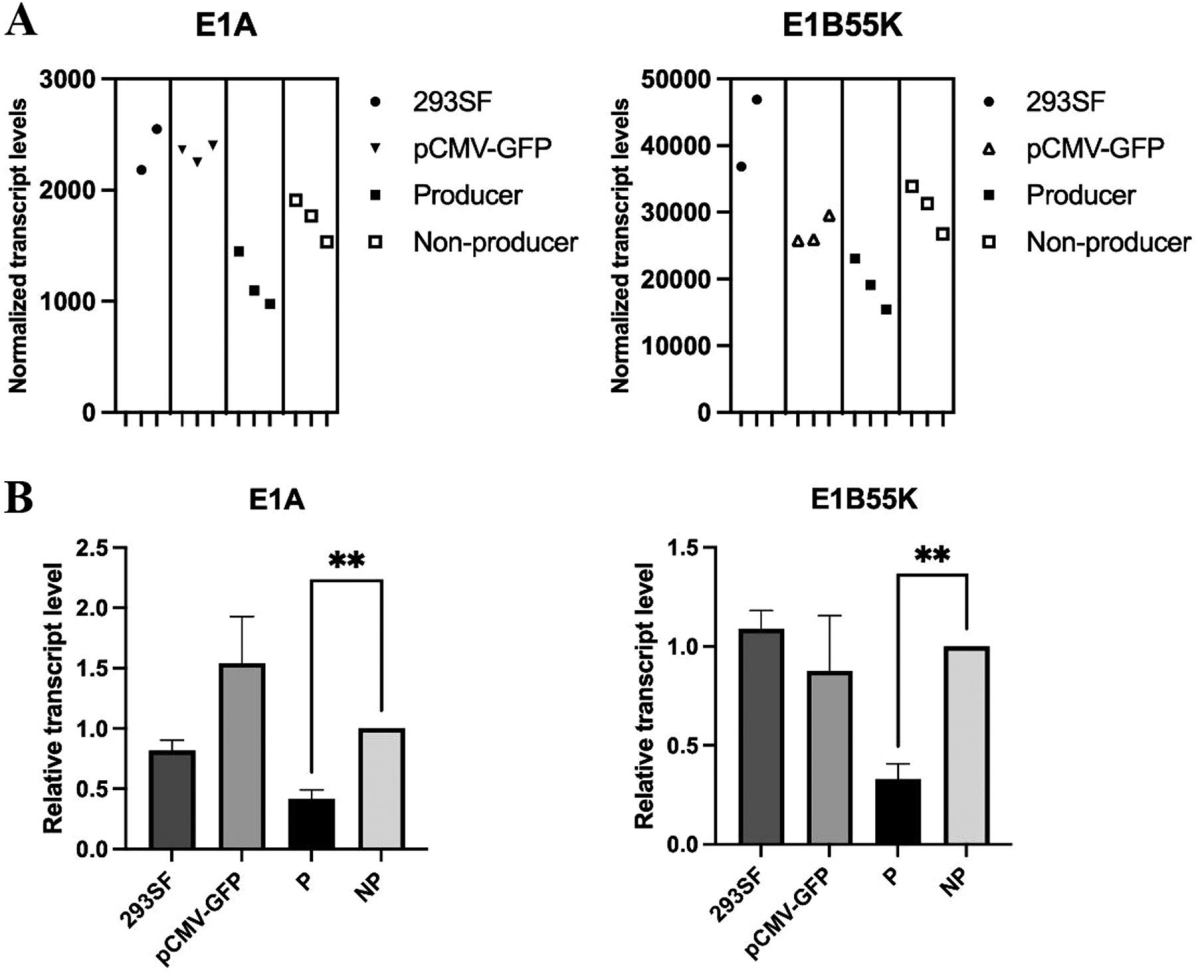
Expression of Ad‐helper E1A and E155K. A. RNASeq analysis of Ad‐helper E1A and E1B55K. Cells were harvested from a triple transfection for AAV‐production. After fixation, permeabilization and staining as described in Materials and Methods, the populations GFP^+^AF^−^ (non‐producer) and GFP^+^AF^+^ (producer) were collected using flow cytometry. Similarly, cells were harvested, stained and sorted to isolate the GFP^+^ population in the pCMV‐GFP transfection. 293SF cells were also fixed, stained and permeabilized. Total RNA was extracted and sequenced as described in Materials and Methods. The figure represents the average of normalized transcript levels from three independent experiments. B. RT‐ddPCR analysis of Ad‐helper E1A and E1B55K: RNA was subjected to RT‐ddPCR analysis as described in Materials and Methods. The data was normalized to the GFP^+^AF^−^ sample within the experiment. The figure represents the average +/− s.d. of normalized transcript levels from three independent experiments. Graph pad prism was used to plot data and perform t‐tests to determine statistical significance. ** *p* < 0.01.

In addition to the adenoviral helper genes that are transfected, the adenoviral genes, E1A and E1B are expressed constitutively in the HEK293 cells. Although they play critical roles in supporting the AAV life cycle, they also interfere with the function of the key AAV regulatory protein Rep 78 (Saudan 2000). Comparison of normalized transcript levels revealed lower levels of both E1A and E1B55K in the AAV‐producer population compared to 293SF, pCMV‐GFP and non‐producer populations (Figure 2A). These results were confirmed by RT‐ddPCR in three independent experiments (Figure 2B).

### 3.3 Cell Cycle Analysis

**Figure 3:**
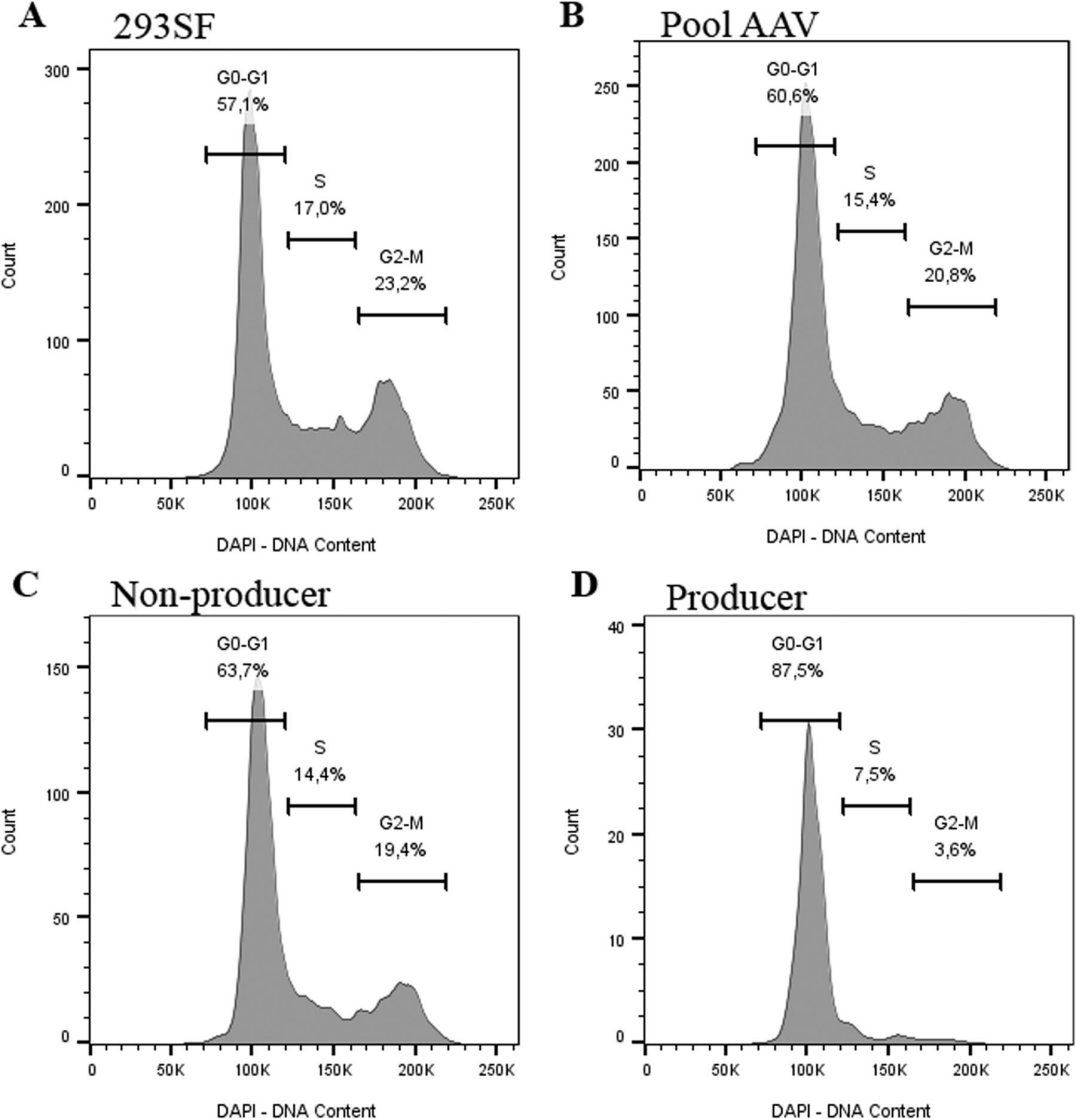
Cell cycle profile of AAV‐producing and nonproducing cell populations. Un‐transfected 293SF and cells transfected to produce AAV were fixed and stained with A20R‐AF568. DAPI was added before analysis by flow cytometry. The figure represents the cell cycle profile of (A) un‐transfected 293SF cells, (B) transfected pool (C) GFP^+^AF^−^ non‐producer sub‐population and (D) GFP^+^AF^+^ producer sub‐population 24 h posttransfection.

Given that one of the key roles of E1A is to promote cell cycle progression (Frisch and Mymryk 2002), we wanted to determine the effect of lowered E1A expression on the cell cycle profile of the two cell populations, producer and non‐producer. Transfected cells were fixed, permeabilized and stained with A20R‐AF568 and DAPI as described in the Materials and Methods section and analyzed by flow cytometry. As seen in Figure 3A–C, there is no significant difference between the cell cycle profiles of 293SF, transfected pool or non‐producer cells. However, the producer population, shown in Figure 3D displays a distinctly different profile. The percentage of cells in the G1/G0 phase of the cell cycle are 57.1, 60.6, and 63.7 for un‐transfected, transfected pool and non‐producers, respectively. In the producer population (Panel D) G1/G0 fraction represents 87.5% of cell population. Similarly, the S and G2/M phase in the producers is 7.5% and 3.6% as opposed to 17.0% and 23.2%, 15.4% and 20.8% and 14.4% and 19.4% in the un‐transfected, transfected pool and non‐producers, respectively.

### 3.4 Expression of Cellular Genes

**Figure 4:**
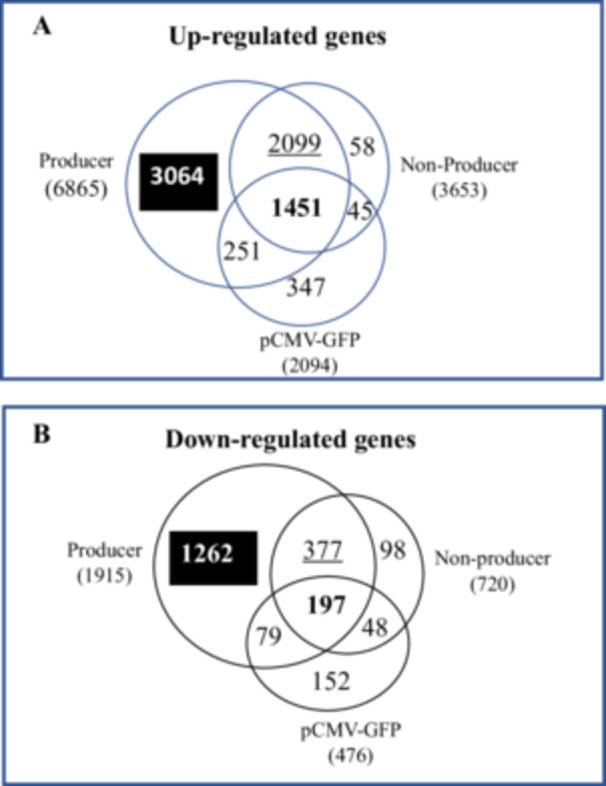
Venn diagram of differentially expressed transcripts. Transcripts expressed in producer, non‐producer and pCMV5‐GFP cell populations were compared to those in the 293SF. The Figure shows the transcripts that are shared between the different cell populations for the A. Upregulated and B. Downregulated transcripts. Differentially regulated transcripts for the 3 cell populations were subjected to GSEA analysis and the enriched pathways are tabulated in Table S2.

To identify genes linked to these functional differences, we examined changes in gene expression profiles as a response to transfection and to the expression of viral genes. Figure 4 shows Venn diagrams representing the changes in transcript levels with respect to 293SF with an increase or decrease of a twofold change (Log2 ≥ 1 or ≤ −1), with *p* value ≤ 0.01. It can be seen that there are 1451 (A) transcripts that are upregulated and 197 (B) that are downregulated in all 3 populations, AAV‐producer, non‐producer and pCMV‐GFP transfection. A total of 6865 transcripts are upregulated and 1915 are downregulated in the AAV‐producer population, many of which (2099 upregulated and 377 downregulated) are similarly regulated in the non‐producer population. Finally, this data reveals the contribution of transfection per se, since 330 (251 upregulated and 79 downregulated) transcripts contribute to the profile of producers, while 93 (45 upregulated and 48 downregulated) contribute to that of the non‐producer population.

### 3.5 Transcriptomic Profile of Producers

**Figure 5:**
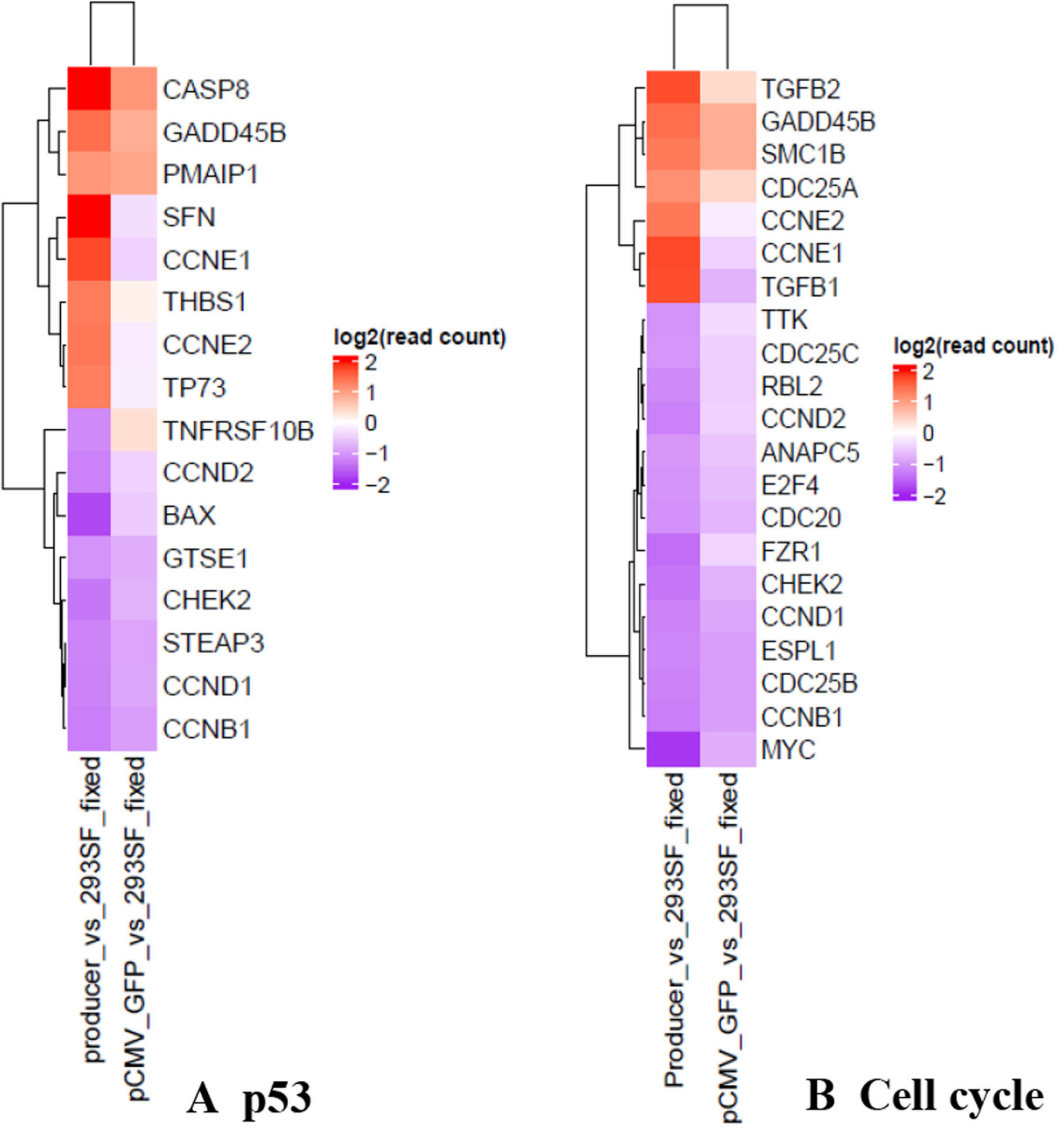
Transcripts contributing to pathways enriched in the producer population. Differentially regulated transcripts (producer vs 293SF and pCMV‐GFP vs 293SF) were subjected to GSEA analysis. The downregulation of the p53 and cell cycle pathways, emerged as statistically significant in the producer population (*p* ≤ 0.05). (Table S2) The figure shows a heat map representing differentially regulated transcripts contributing to these pathways in the producer population. The relative levels of the same transcripts in the CMV‐GFP population are presented for comparison. A. p53 pathway: The relative levels of transcripts in the producer and pCMV‐GFP samples, both compared to those in 293SF are represented in a heatmap. Up/downregulation is not statistically significant (*p* > 0.01) in the pCMV‐GFP sample for SFN (*p* = 0.59), CCNE2 (*p* = 0.21), THBS1 (*p* = 0.69) and TP73 (*p* = 0.22). See also Figure S2A. B. Cell cycle: The relative levels of transcripts in the producer and pCMV‐GFP samples, both compared to those in 293SF are represented in a heatmap. Up/downregulation is not statistically significant (*p* > 0.01) in the pCMV‐GFP sample for TGFB2 (*p* = 0.23), CCNE2 (*p* = 0.21) and SMC1B (*p* = 0.015). See also Figure S2B.

The differentially‐regulated list of genes was subjected to Gene Set Enrichment Analysis (GSEA)(Mootha et al. 2003; Subramanian et al. 2005) to investigate the gene association with KEGG pathways. A few pathways that emerged as significantly enriched (*p* ≤ 0.05) were p53 signaling, cell cycle, lysosome, peroxisome and gap junction, all being downregulated (Table S2). Examination of the transcripts involved in the p53 pathway, reveals an increased transcript level of a member of the p53 family of tumor suppressors, TP73 (Kong et al. 2023), and targets of p53 (Bieging et al. 2014) such as apoptosis‐inducing protein SFN and the growth arrest and DNA damage‐induced GADD45B (Figures 5A and S2A). These changes are indicative of a response to DNA‐damage, which in turn, is known to trigger a block in cell cycle progression. Moreover, upregulation of THBS1(Gutierrez and Gutierrez 2021) has been shown to activate latent TGFB1, a key negative regulator of the cell cycle (Kubiczkova et al. 2012). Indeed, there is an upregulation of the TGFB1 transcript in the producer population, accompanied by the downregulation of MYC, E2F4, CCND1, CCND2, CCNB1, all of which point to a block in the cell cycle (Cell cycle Figures 5B and S2B). The downregulation of the peroxisome and lysosome pathways is also a reflection of the absence of normal cell proliferation (Nowosad and Besson 2022; Yan et al. 2005). AAV benefits from a G1/S arrest as the DDR enzyme polδ is activated during this phase and has been implicated in the replication of the AAV genome (Groelly et al. 2023; Ning et al. 2023). The ability of Rep78 to block cells in the S‐phase, without cellular DNA synthesis, has been reported earlier (Berthet et al. 2005). In the absence of DNA synthesis, the cells appear in G1 when DNA content is used to assign the cell cycle phase, but the increased expression of Cyclins E1 and E2 would suggest presence in the S‐phase (Figures 5B and S2B). Although this is an apparent contradiction, the ambiguity stems from the atypical nature of the cell cycle block imposed by AAV‐Rep, that is, an S‐phase without the key activity that characterizes this phase, replication of genomic DNA.

The crucial role of cell cycle regulation in various aspects of AAV production has been demonstrated in several studies. An elegant visualization of intracellular DNA post‐transfection was reported by Budge (Budge 2025), who used fluorescently‐labelled DNA and showed the importance of nuclear envelope breakdown for DNA entry. Upon entry, subsequent dilution of DNA can be prevented by the addition of agents such as HMN‐214, that inhibit the transition of HEK293 cells from G2/M to G1. This block in cell cycle progression results in an increase in nuclear DNA and as a consequence, functional AAV titers (Fisher et al. 2025)

Particularly challenging in AAV production is the large amounts of capsid proteins required for viral particle formation. We have shown that transfected cells unable to produce AAV, are lacking primarily in capsid expression (Figure 1). Of note, this sub‐population is not blocked from cell cycle progression, in contrast to the producer population, that synthesizes significantly higher amounts of capsid proteins (Figure 3). Reducing the proliferative capacity of cells to divert energy for protein synthesis was first reported in a pioneering study by Fussenegger et al (Fussenegger et al. 1997). Since then several strategies have been implemented to disrupt the cell cycle to improve productivity including both chemical and cell engineering approaches (Donaldson et al. 2021). Focussing more specifically on the production of AAV, numerous studies have described the crucial role of cell cycle in AAV biogenesis. For instance, Barnes and colleagues (Barnes et al. 2021), carried out a genome wide CRISPR activation screen to identify cellular factors that could increase AAV productivity and found gain‐of‐function targets, SKA2 and ITPRIP, both implicated in cell cycle regulation. Moreover, they demonstrated that cells blocked from cell cycle progression by the addition of Thymidine, a DNA synthesis inhibitor that can arrest cell at G1/S boundary (Chen and Deng 2018), were able to produce higher titers of full capsids. Another group that carried out an extensive time course analysis of the changes in gene expression profiles during AAV production reported on an overwhelming inflammatory response (Chung et al. 2023) Such a response is often observed as a reaction to foreign DNA (Warga et al. 2023) and in the absence of a mock transfection, it is difficult to discriminate between transfection‐driven and AAV‐driven responses.

**Figure 6:**
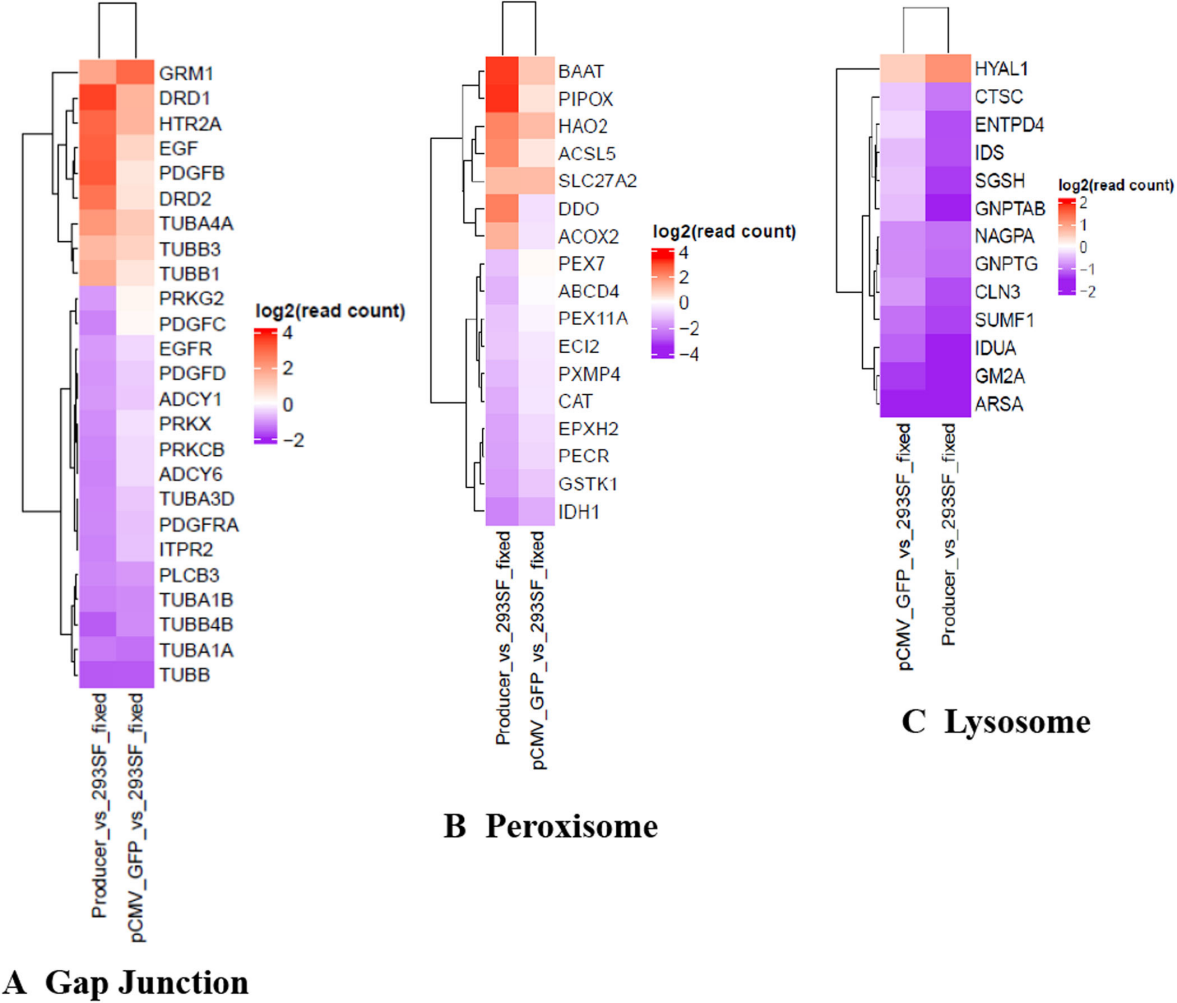
Transcripts contributing to pathways enriched in the producer population: Differentially regulated transcripts (producer vs 293SF and pCMV‐GFP vs 293SF) were subjected to GSEA analysis. The downregulation of gap junction, peroxisome and lysosome pathways emerged as statistically significant in the producer population (*p* ≤ 0.05). (Table S2) The figure shows a heat map representing differentially regulated transcripts contributing to these pathways in the producer population. The relative levels of the same transcripts in the pCMV‐GFP population are presented for comparison. A. Gap junction: The relative levels of transcripts in the producer and pCMV‐GFP samples, both compared to those in 293SF are represented in a heatmap. Up/downregulation is not statistically significant (*p* > 0.01) in the pCMV‐GFP transfected cells for DRD1 (*p* = 0.09), PDGFB (*p* = 0.39), EGF (*p* = 0.026), HTR2A (*p* = 0.17), DRD2 (*p* = 0.19), TUBA4A (*p* = 0.018), TUBB1 (*p* = 0.08), PRKG2 (*p* = 0.4), PDGFD (*p* = 0.098), PDGFRA (*p* = 0.069), PRKCB (*p* = 0.12), TUBA3D (*p* = 0.02) and PDGFC (*p* = 0.49). See also Figure S2C. B. Peroxisome: The relative levels of transcripts in the producer and pCMV‐GFP samples, both, compared to those in 293SF are represented in a heatmap. Up/downregulation is not statistically significant (*p* > 0.01) in the pCMV‐GFP sample for PIPOX (*p* = 0.11), BAAT (*p* = 0.02), DDO (*p* = 0.57), HAO2 (*p* = 0.04), ACSL5 (*p* = 0.24), ACOX2 (*p* = 0.33), EC12 (*p* = 0.017), PEX11A (*p* = 0.3), PEX7 (*p* = 0.59), PXMP4 (*p* = 0.02), ABCD4 (*p* = 0.52). See also Figure S2D. C. Lysosome: The relative levels of transcripts in the producer and pCMV‐GFP samples, both, compared to those in 293SF are represented in a heatmap. Up/downregulation is not statistically significant (*p* > 0.01) in the pCMV‐GFP sample for HYAL1 (*p* = 0.14), IDS (*p* = 0.04) and CLN3 (*p* = 0.03). See also Figure S2E.

As opposed to the pathways discussed above that are downregulated only in the producer population, the odorant receptor pathway is upregulated in both the AAV‐producer and non‐producer populations. The change in transcript levels for most of the genes appears to be a response to viral proteins, since very few show a statistically significant response in the pCMV‐GFP transfection (Figures S3A and S3B). While the enrichment of the pathways is statistically significant in both producers and non‐producers, the biological significance of this observation is unclear, since the level of expression is very low (5/24 and 6/44 transcripts with a log base mean value < 20 in non‐producers and producers respectively). Although, the ectopic expression and activation of certain olfactory receptors have been ascribed roles in several cellular processes (Maßberg and Hatt 2018) including malignancies (Kalra et al. 2020), without further validation, it is difficult to know whether these changes have a functional consequence in this model. Figure 6.

**Figure 7:**
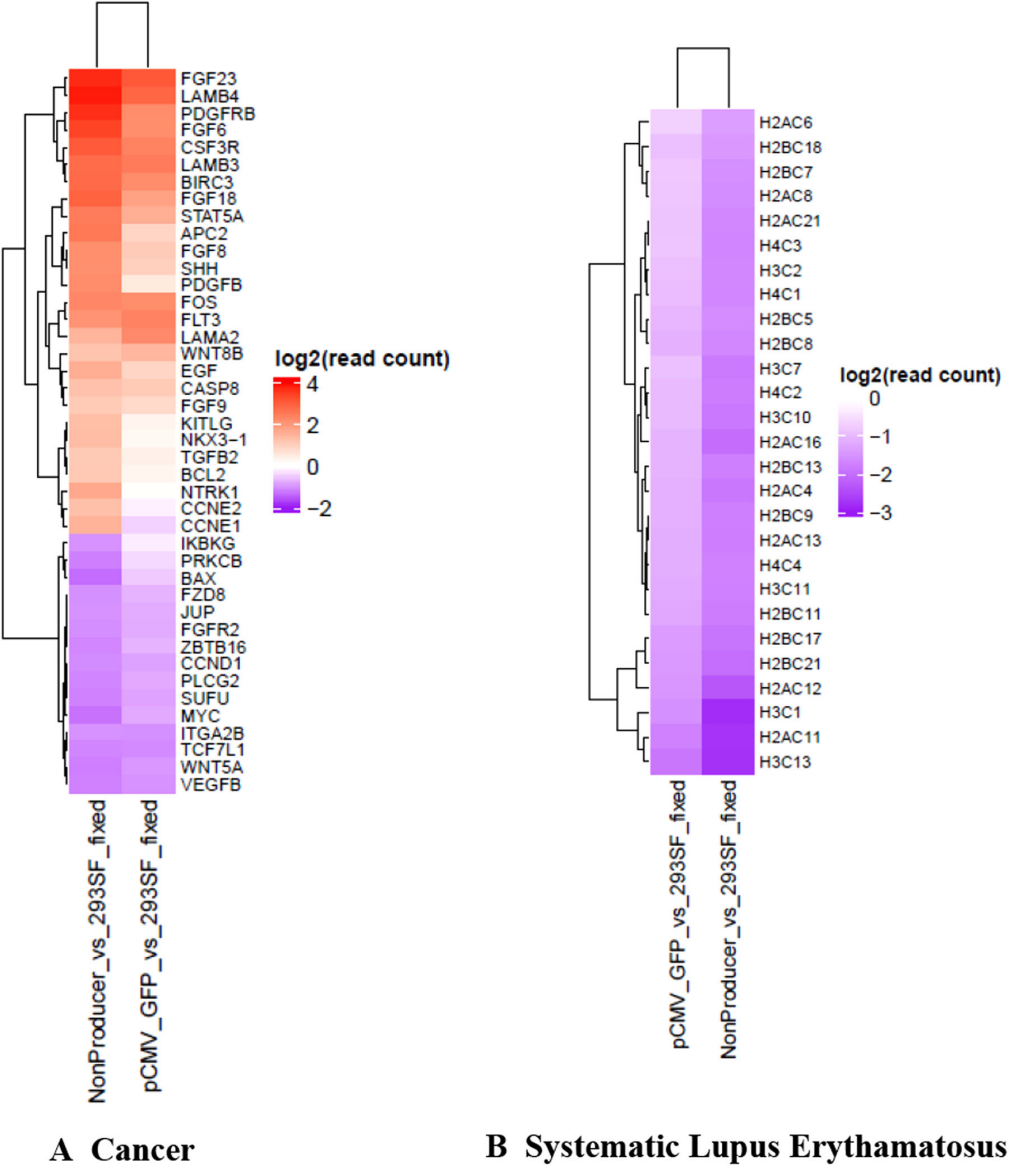
Transcripts contributing to pathways enriched in the non‐producer population: Differentially regulated transcripts (non‐producer vs 293SF and pCMV‐GFP vs 293SF) were subjected to GSEA analysis. The downregulation of the cancer and Systemic Lupus Erythematosus (SLE) pathways emerged as statistically significant in the non‐producer population (*p* ≤ 0.05). (Table S2) The figure shows a heat map representing differentially regulated transcripts contributing to these pathways in the non‐producer population. The relative levels of the same transcripts in the pCMV‐GFP population are presented for comparison. A. Cancer The relative levels of transcripts in the non‐producer and pCMV‐GFP samples, both compared to those in 293SF are represented in a heatmap. Up/downregulation is not statistically significant (*p* > 0.01) in the pCMV‐GFP sample for PDGFRB (*p* = 0.05), FGF6 (*p* = 0.02), PDGFB (*p* = 0.39), FGF8 (*p* = 0.049), SHH (*p* = 0.03), NTRK1 (*p* = 0.6), EGF (*p* = 0.026), NKX3‐1 (*p* = 0.25), KITLG (*p* = 0.02), CCNE2 (*p* = 0.21), TGFB2 (*p* = 0.23) and IKBKG (*p* = 0.36). See also Figure S3A. B. Systemic Lupus Erythamatosus The relative levels of transcripts in the non‐producer and pCMV‐GFP samples, both compared to those in 293SF are represented in a heatmap. See also Figure S3B.

**What is different about non‐producers?** 3653 and 720 genes were up and downregulated respectively, in the non‐producer population in comparison to 293SF (Figure 4). This gene list was subjected to a GSEA analysis that revealed 3 downregulated pathways, (Cancer, Systemic Lupus Erythamatosus (SLE) and Neurotrophin) that were statistically significant. (Cancer p = 0, SLE p = 0 and Neurotrophin p = 0 and Table S2). Examining the list of transcripts implicated in the Cancer pathway, an upregulation of a proliferative stimulus (FGF6, FGF8, FGF9, FGF8, FGF23, FOS, PDGFB, EGF, KITLG) is evident (Figures 7A and S4A). Although several transcripts for histones are downregulated in the non‐producers (Figure S4B), the SLE pathway appears to be a response to transfection per se, given that the changes are also statistically significant for all transcripts in the pCMV‐GFP transfected population.

**Figure 8:**
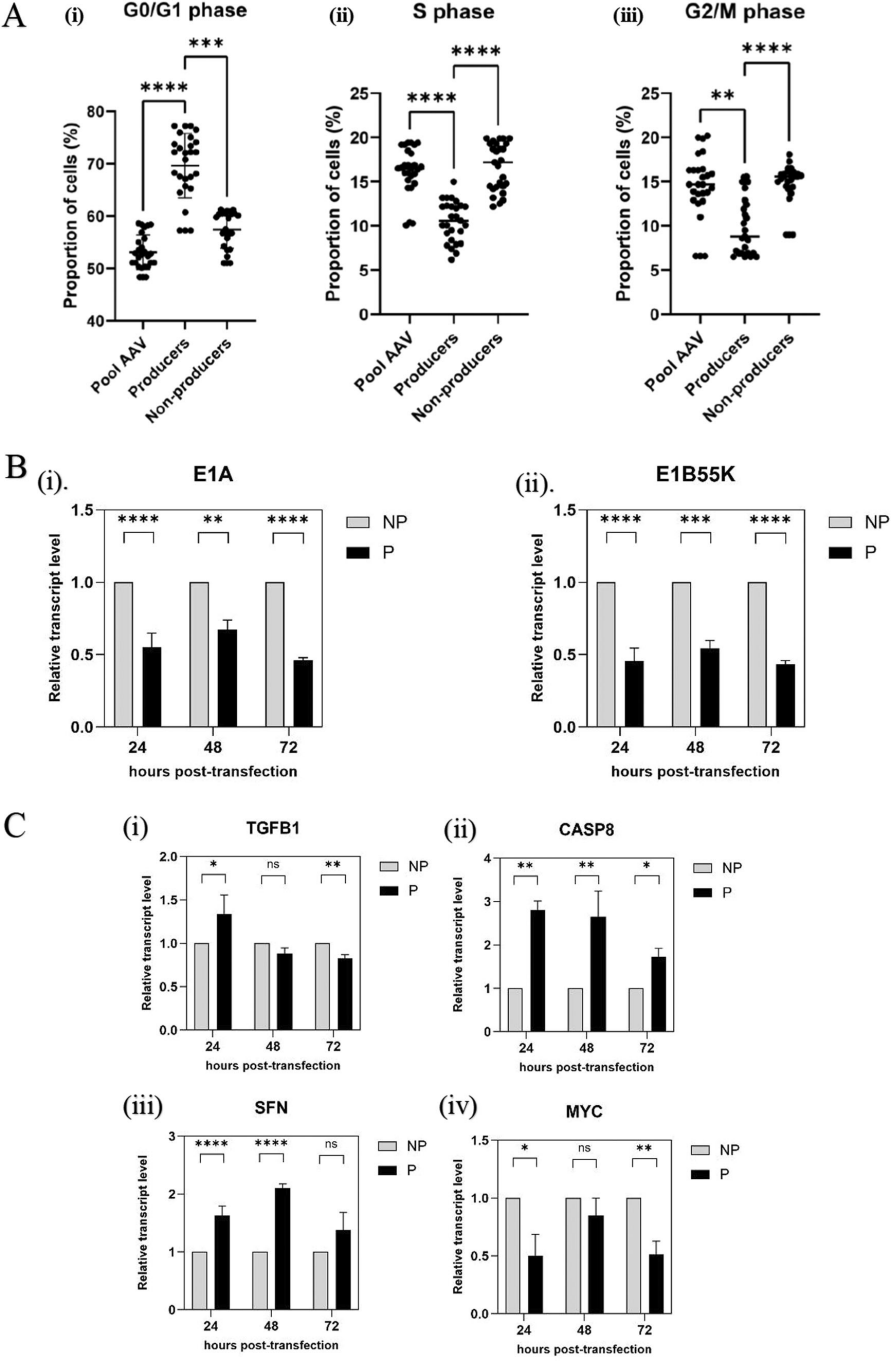
Comparison of Producer and Non‐producer populations over 72 h of AAV production. A. Cell cycle profile Cells transfected to produce AAV were fixed and stained with A20R‐AF568. DAPI was added before analysis by flow cytometry. The figure shows the percentage of cells in the G0/G1, S and G2/M phases of the cell cycle, combining the results of 3 different time points, 24, 48 and 72 h posttransfection. B. and C. q‐PCR RNA was subjected to RT‐qPCR analysis as described in Materials and Methods. The data was normalized to the GFP^+^AF^−^ sample within the experiment. The figure represents the average +/‐ standard deviation (s.d.) of relative transcript levels from three independent experiments. Graph pad prism was used to plot data and perform ANOVA to determine statistical significance. *p < 0.05, **p < 0.01, ***p < 0.001 and **** *p* < 0.0001. B. Adenoviral helper genes (i) E1A and (ii) E1B55K **C.** Cellular genes (i) TGFB1 (ii) CASP8 (iii) SFN and (iv) MYC.

Having established that cells producing AAV are blocked from cell cycle progression linked to a downregulation of E1A and E1B55K and significant alterations in cell cycle and p53 pathways, 24 h post‐transfection, we wanted to extend our findings during 72 h of production. Figure 8 shows the results of analysis carried out over this extended period. Figure 8A,B show that a block in cell cycle progression and the decrease in adenoviral helper gene expression is also observed in AAV‐producer cells, 48 and 72 h post transfection. Panel C shows the validation of changes in transcript levels for key genes implicated in cell cycle control identified in the RNAseq analysis. As expected, 24 h posttransfection, expression of TGFB1, SFN and CASP8 is higher and MYC is lower, in AAV‐producers compared to non‐producers. Extending the analysis in time, we see that, targets of TGFB1 such as SFN and CASP8 remain higher in producers compared to non‐producers 48 and 72 h posttransfection. MYC, on the other hand, remains lower in producers, consistent with the cell cycle block in the producer population. Given the antagonistic relationship between the E1A and the TGFB1 pathways, lower levels of E1A and E1B, are consistent with higher levels of TGFB1 and its targets and consequently, a block in cell cycle. Thus, our data has provided a unique insight into the molecular processes required for AAV biogenesis. It is interesting to speculate that regulating the relative amounts of Rep and Ad helper proteins, E1A and E1B55K, may be key to high productivity.

## Author Contributions

Audrey Morasse, Melanie Leclerc, Annie Viau, Sonia Leclerc, and Milica Momcilovic conducted the experiments. Ziying Liu and Qing Yan Liu carried out analysis of RNA‐seq data. Alaka Mullick and Amine A. Kamen wrote the manuscript.

## Conflicts of Interest

The authors declare no conflicts of interest.

## Supporting information


**Table S1:** Primers for PCR. **Table S2:** Pathway enrichment. **Figure S1:** Plasmid maps of transfected DNA. **Figure S2:** Pathways significantly enriched in producer population. **Figure S2B:** Cell cycle. **Figure S2C:** Gap junction. **Figure S2D:** Peroxisome. **Figure S2E:** Lysosome. **Figure S3:** Olfactory transduction. **Figure S3B:** Producer. **Figure S4:** Pathways significantly enriched in the non‐producer population. **Figure S4B:** Systemic Lupus Erythamatosus (SLE).

## Data Availability

Bulk RNA‐seq data are accessible through GEO via accession number (GSE276141). https://www.ncbi.nlm.nih.gov/geo/query/acc.cgi?acc=GSE276141.
