# Supplementary material for: AAV Assembled Capsids Are Produced in Cells Blocked From Cell Cycle Progression
Source: Biotechnol Bioeng. 2025 Nov 18;123(2):273–86. doi: 10.1002/bit.70111 (PMC12779224; doi:10.1002/bit.70111)
Supplement: Supplementary file 1 — Table S1: Primers for PCR. Table S2: Pathway enrichment. Figure S1: Plasmid maps of transfected DNA. Figure S2: Pathways significantly enriched in producer population. Figure S2B: Cell cycle. Figure S2C: Gap junction. Figure S2D: Peroxisome. Figure S2E: Lysosome. Figure S3: Olfactory transduction. Figure S3B: Producer. Figure S4: Pathways significantly enriched in the non‐producer population. Figure S4B: Systemic Lupus Erythamatosus (SLE). [file BIT-123-273-s001.docx]

**Table S1. Primers for PCR**

|  | **Producer** | | **Non-producer** | | |
| --- | --- | --- | --- | --- | --- |
| **Down-regulated** | number of genes | Nominal p-value | | Number of genes | Nominal p-value |
| p53 | 17 | 0 | |  |  |
| Cell cycle | 23 | 0 | |  |  |
| Peroxisome | 17 | 0 | |  |  |
| Lysosome | 17 | 0.033 | |  |  |
| Gap junction | 26 | 0 | |  |  |
| Neurotrophin | 22 | 0.75 | | 15 | 0 |
| Cancer | 78 |  | | 44 | 0 |
| SLE | 63 |  | | 50 | 0 |
| **Up-regulated** |  |  | |  |  |
| Olfactory transduction | 64 | 0 | | 35 | 0.001 |

**Table S2: Pathway enrichment:** Transcript profiles of Producer, Non-Producer and pCMV-GFP populations were compared to those of 293SF. Differentially-regulated genes were subjected to GSEA analysis. The Table shows the pathways that were significantly enriched (p ≤ 0.05)


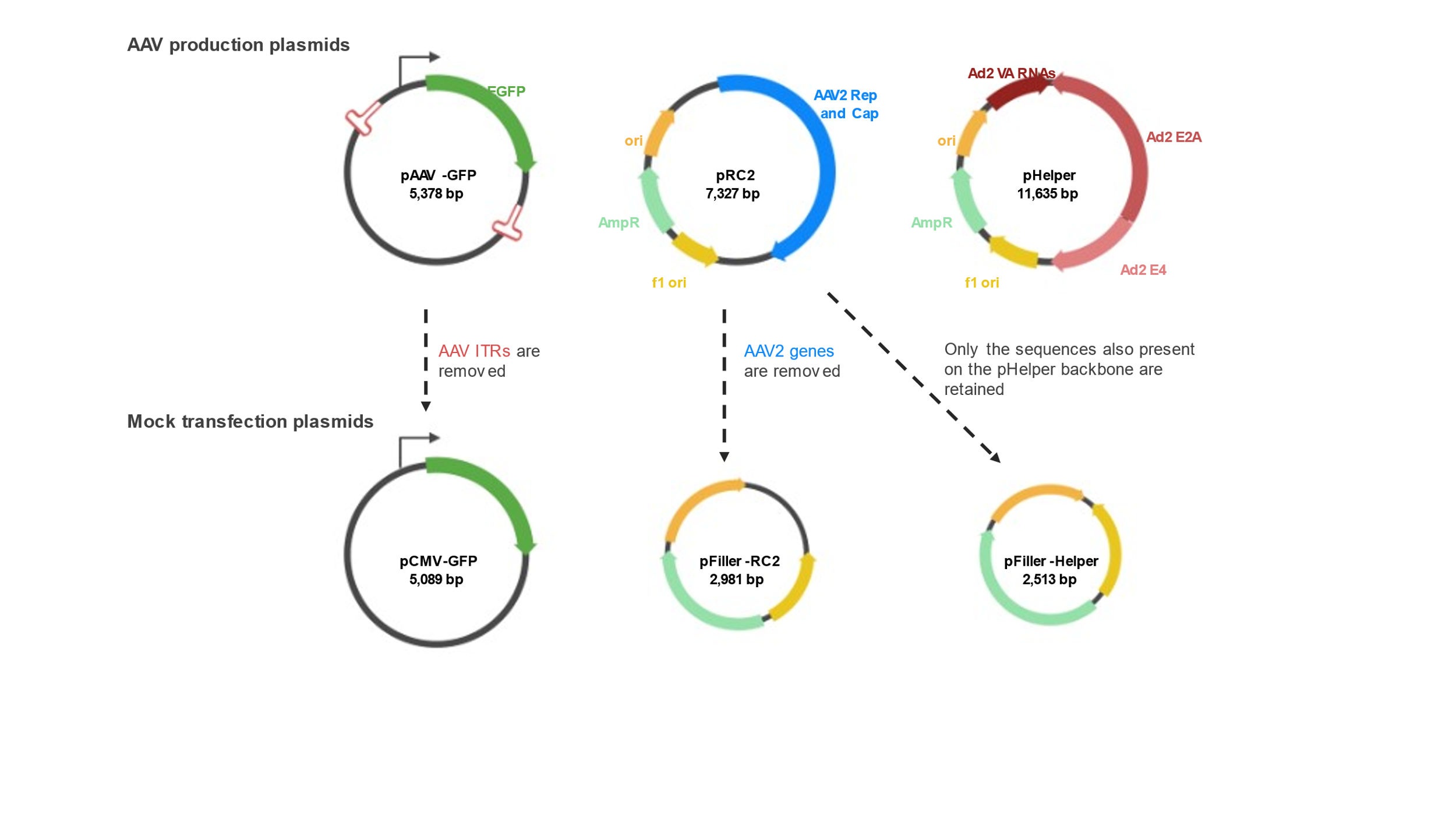


**Figure S1 Plasmid maps of transfected DNA**


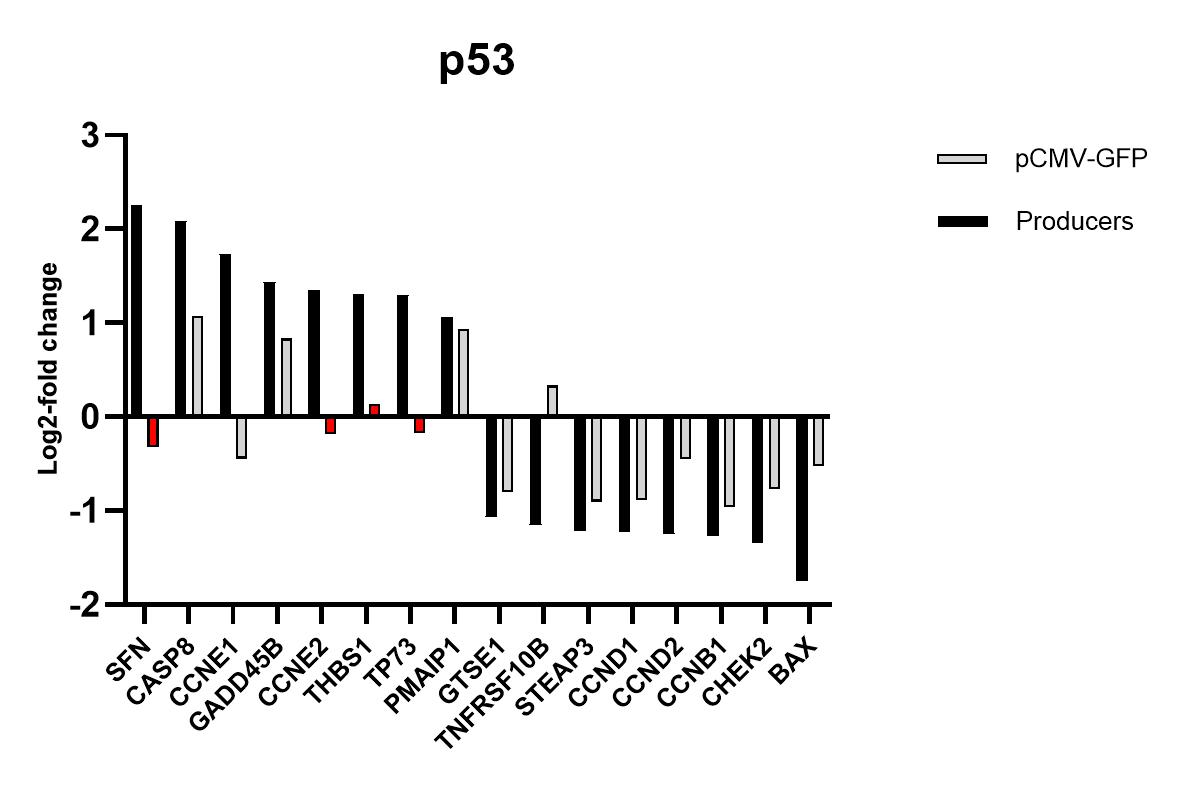


**Figure S2. Pathways significantly enriched in producer population:** Differentially regulated transcripts (producer vs 293SF and pCMV-GFP vs 293SF) were subjected to GSEA analysis. The down-regulation of the p53 and cell cycle pathways, emerged as statistically significant in the producer population (p ≤ 0.05). (Table S2) The figure shows relative levels of transcripts contributing to these pathways in the producer population. The relative levels of the same transcripts in the pCMV-GFP population are presented for comparison. **A p53 pathway:** The figure shows relative levels of transcripts in the producer and pCMV-GFP samples, both compared to transcript levels in 293SF. Up-regulation is not statistically significant (p > 0.01) in the pCMV-GFP sample for SFN (p = 0.59), CCNE2 (p = 0.21), THBS (p = 0.69) and TP73 (p = 0.22) represented by red bars.


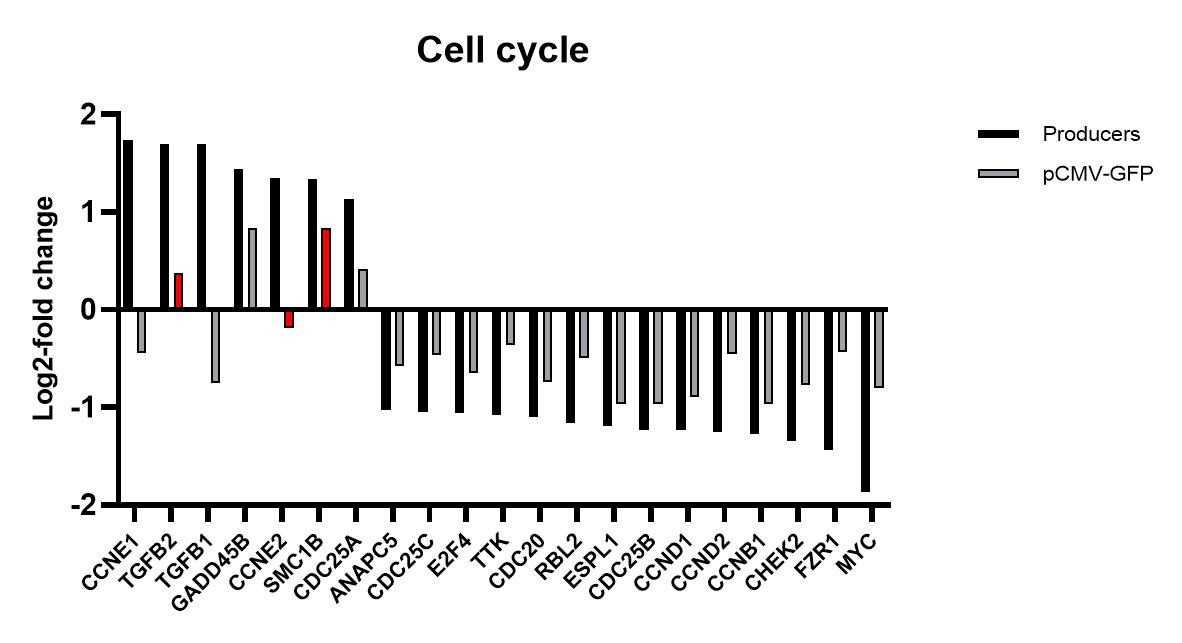


**Figure S2B. Cell cycle:** The figure shows relative levels of transcripts in the producer and pCMV-GFP samples, both compared to transcript levels in 293SF. Up/down-regulation is not statistically significant (p > 0.01) in the pCMV-GFP sample for TGFB2 (p = 0.23), CCNE2 (p = 0.21) and SMC1B (p = 0.015) represented by red bars.


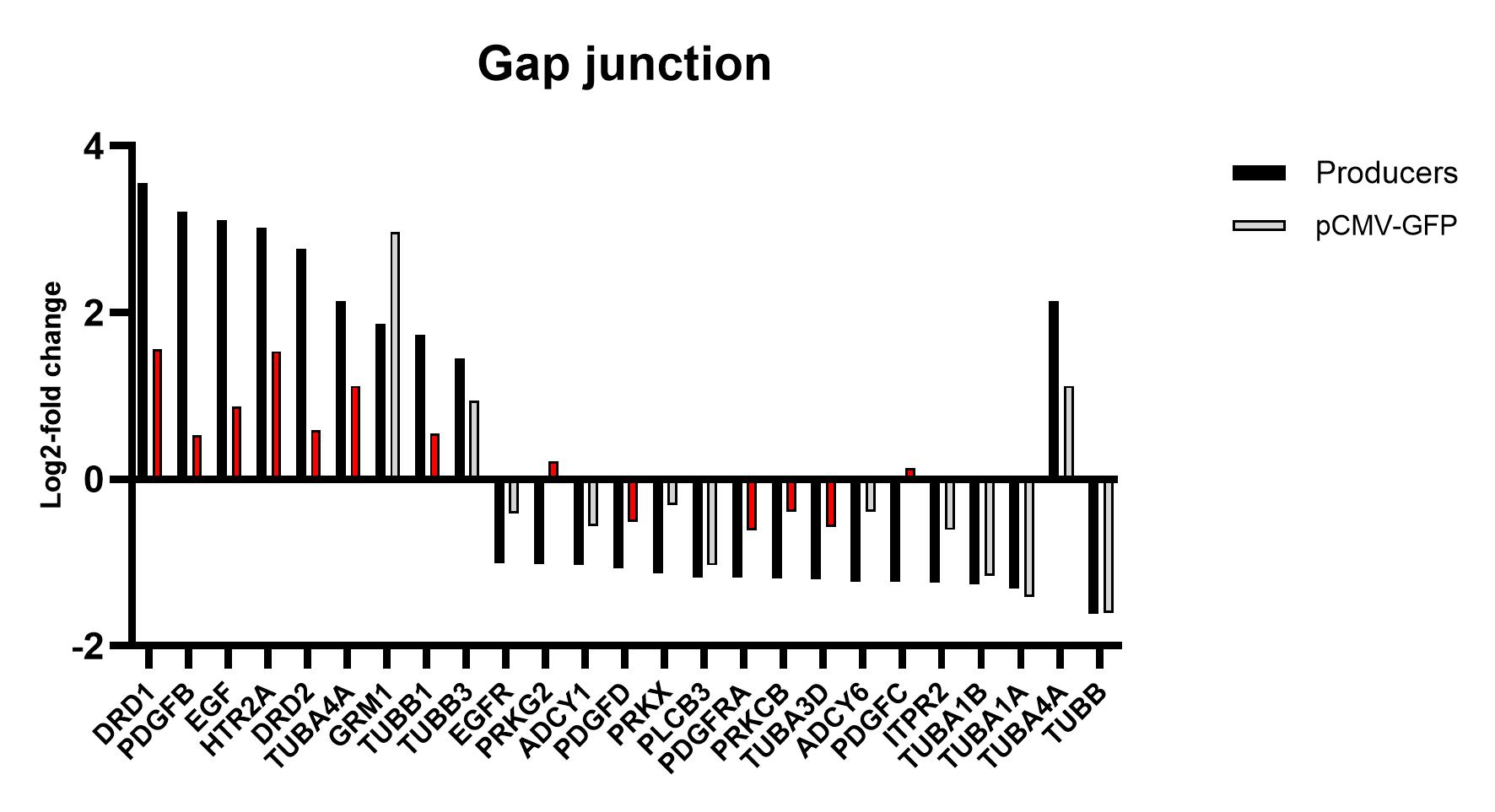


**Figure S2C. Gap junction:** The figure shows relative levels of transcripts in the producer and pCMV-GFP samples, both compared to those in 293SF. Up/down-regulation is not statistically significant (p > 0.01) in the pCMV-GFP sample for DRD1 (p = 0.09), PDGFB (p = 0.39), EGF (p = 0.026), HTR2A (p = 0.176), DRD2 (p = 0.19), TUBA4A (p = 0.018), TUBB1 (p = 0.08), PRKG2 (p = 0.4), PDGFD (p = 0.098), PDGFRA (p = 0.069), PRKCB (p = 0.12), TUBA3D (p = 0.02) and PDGFC (p = 0.49), represented by red bars.


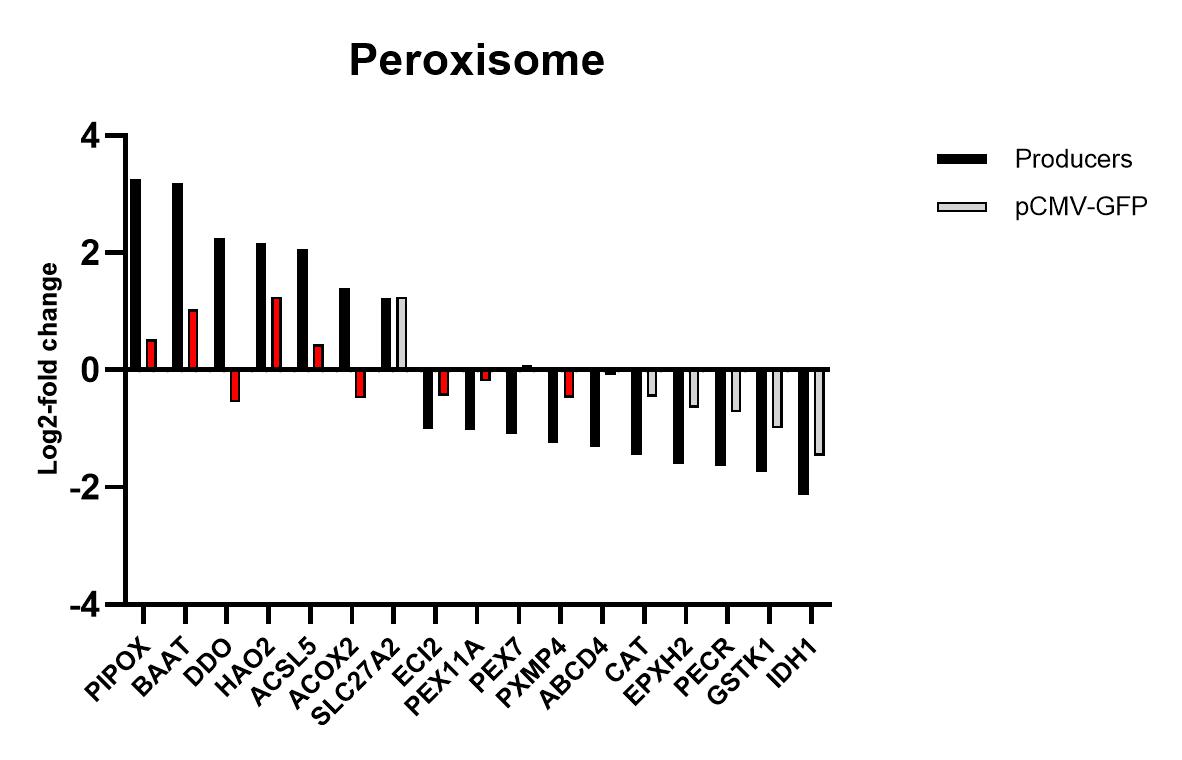


**Figure S2D. Peroxisome:** The figure shows relative levels of transcripts in the producer and pCMV-GFP samples, both compared to those in 293SF. Up/down-regulation is not statistically significant (p >0.01) in the pCMV-GFP transfected cells for PIPOX (p = 0.2), BAAT (p = 0.057), DDO (p = NA), HAO2 (p = 1), ACSL5 (p = 0.38), ACOX2 (p = 0.48), EC12 (p = 0.05), PEX11A (p = 0.45), PEX7 (p = 0.7), PXMP4 (p = 0.06), ABCD4 (p = 0.6), CAT (p = 0.011) represented by red bars.


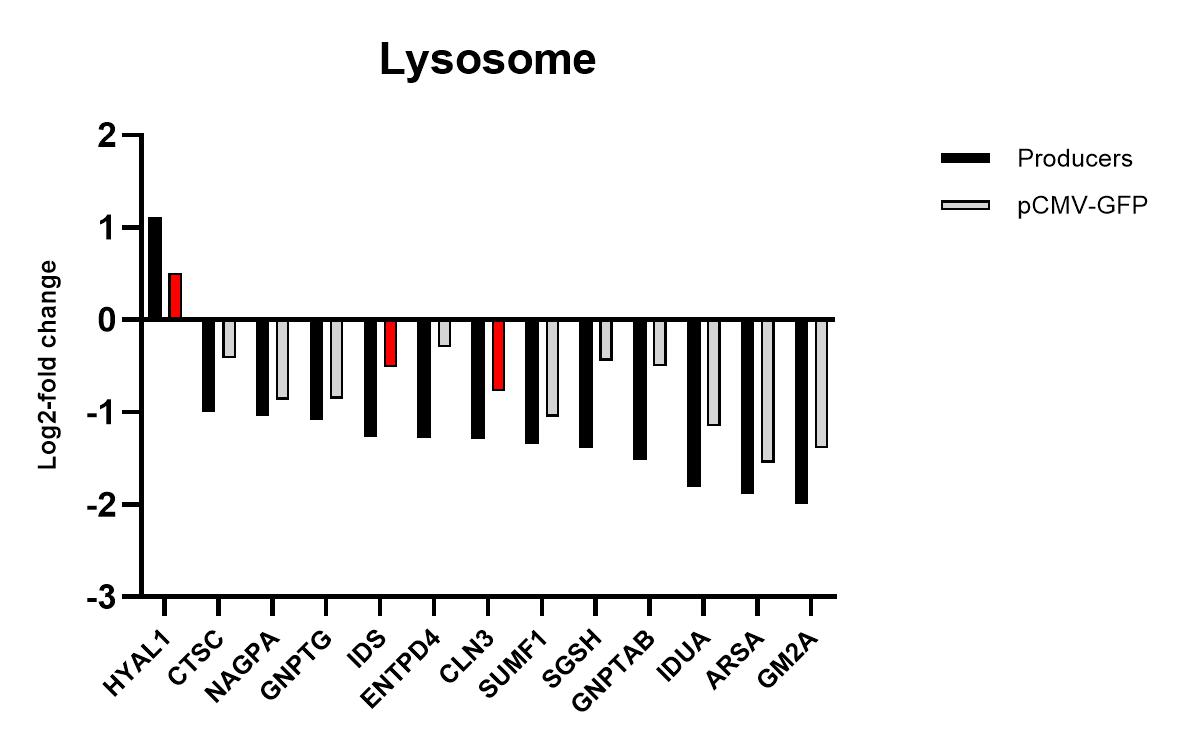


**Figure S2E. Lysosome:** The figure shows relative levels of transcripts in the producer and pCMV-GFP samples, both compared to those in 293SF. Up/down-regulation is not statistically significant (p > 0.01) in the pCMV-GFP sample for HYAL1 (p = 0.14), IDS (p = 0.04) and CLN3 (p = 0.03) represented by red bars


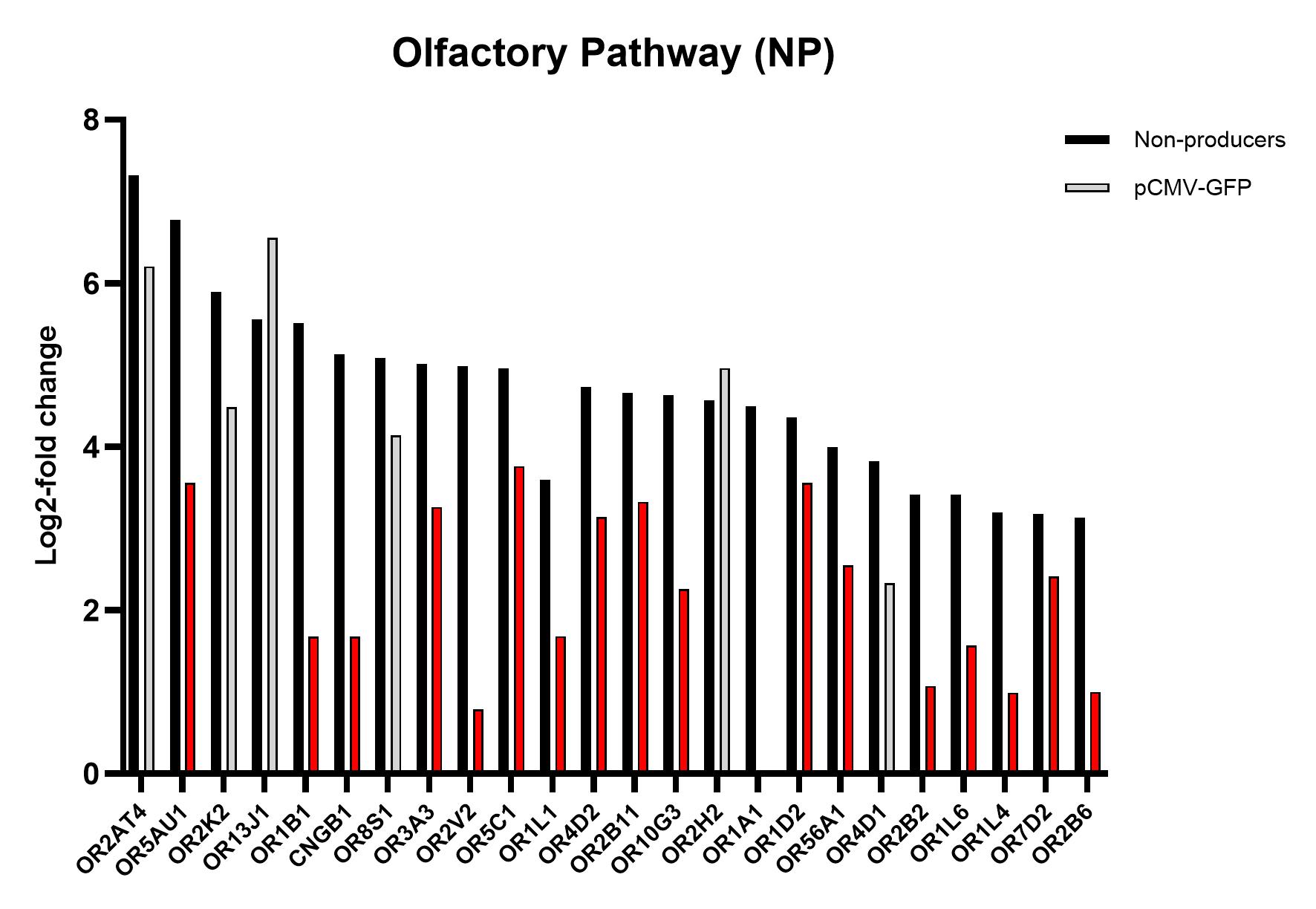


**Figure S3 Olfactory transduction:** Differentially regulated transcripts (producer vs 293SF, non-producer vs 293SF and pCMV-GFP vs 293SF) were subjected to GSEA analysis. The up-regulation of the olfactory transduction pathways emerged as statistically significant in both producer and non-producer population, (p ≤ 0.05). (Table S2) **A. Non producer** The relative levels of transcripts in the non-producer and pCMV-GFP samples compared to those in 293SF. Up-regulation is not statistically significant (p > 0.01) in the pCMV-GFP transfected cells for OR5AU1 (p = 0.016) OR1B1 (p = 0.36), CNGB1 (p = 0.37), OR3A3 (p = 0.047), OR2V2 (p = 0.69), OR5C1 (p = 0.012), OR1L1 (p = 0.39), OR4D2 (p = 0.04), OR2B11 (p = 0.028), OR10G3 (p = 0.21) , OR1A1 (p = 1), OR1D2 (p = 0.02), OR56A1 (p = 0.1), OR2B2 (p = 0.07), OR1L6 (p = 0.19), OR1L4 (p = 0.34), OR7D2 (p = 0.037), OR2B6 (p = 0.098) represented by red bars.


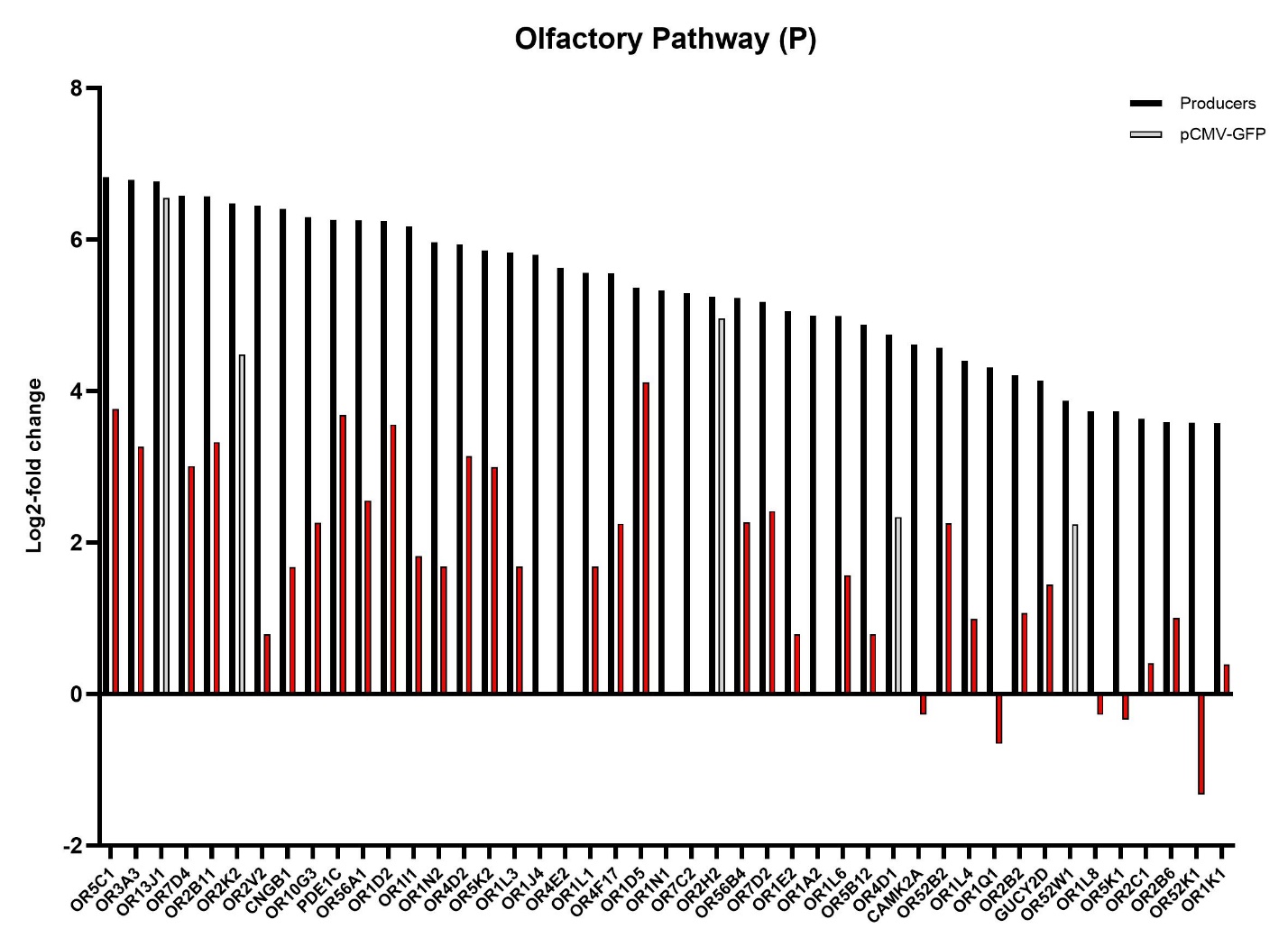


**Figure S3B Producer** The relative levels of transcripts in the producer and pCMV-GFP samples compared to those in 293SF. Up-regulation is not statistically significant (p > 0.01) in the pCMV-GFP-transfected population for OR5C1 (p = 0.012), OR3A3 (p = 0.047), OR7D4 (p = 0.077), OR2B11 (p = 0.028), OR2V2 (p = 0.69), CNGB1 (p = 0.37), OR10G3 (p = 0.21), PDE1C (p = 0.028), OR56A1 (p = 0.1), OR1D2 (p = 0.02), OR1I1 (p = 0.2), OR1N2 (p = 0.38), OR4D2 (p = 0.04), OR5K2 (p = 0.08), OR1L3 (p = 0.39), OR1J4 (p = 1), OR4E2 (p = 1), OR1L1 (p = 0.39), OR4F17 (p = 0.23), OR1D5 (p = 0.016), OR1N1(p = 1), OR7C2(p = 1), OR56B4 (p = 0.22), OR7D2 (p = 0.037), OR1E2 (p = 0.7), OR1A2 (p = 1), OR1L6 (p = 0.19), OR5B12 (p = 0.7), CAMK2A (p = 0.67), OR52B2 (p = 0.06), OR1L4 (p = 0.34), OR1Q1 (p = 0.74), OR2B2 (p = 0.07), GUCY2D (p = 0.07), OR1L8 (p = 0.77), OR5K1 (p = 0.78), OR2C1 (p = 0.68), OR2B6 (p = 0.09), OR52K1 (p = 0.37), OR1K1 ( p = 0.55) represented by red bars.


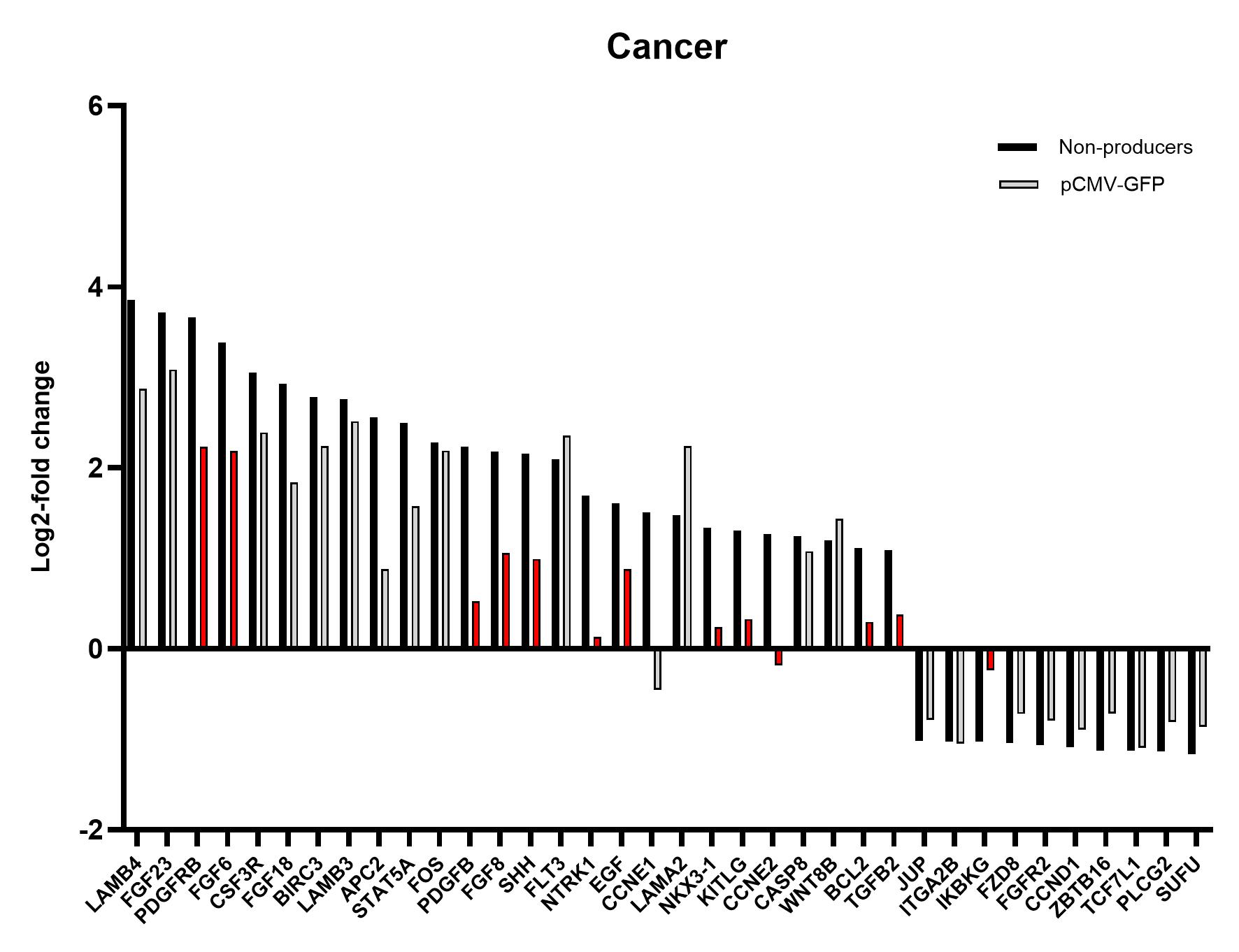


**Figure S4** **Pathways significantly enriched in the non-producer population:** Differentially regulated transcripts (non-producer vs 293SF and pCMV-GFP vs 293SF) were subjected to GSEA analysis. The down-regulation of the cancer and SLE pathways emerged as statistically significant in the non-producer population, (p ≤ 0.05). (Table S2) **A.** **Cancer:** The Figure shows relative levels of transcripts in the non-producer and pCMV-GFP samples compared to those in 293SF. Up/down-regulation is not statistically significant (p > 0.01) in the pCMV-GFP sample for PDGFRB (p = 0.05), FGF6 (p = 0.02), PDGFB (p = 0.39), FGF8 (p = 0.049), SHH (p = 0.03), NTRK1 (p = 0.6), EGF (p = 0.026), NKX3-1 (p = 0.025), KITLG (p = 0.02), CCNE2 (p = 0.2), TGFB2 (p = 0.2) and IKBKG (p = 0.36) represented by red bars.


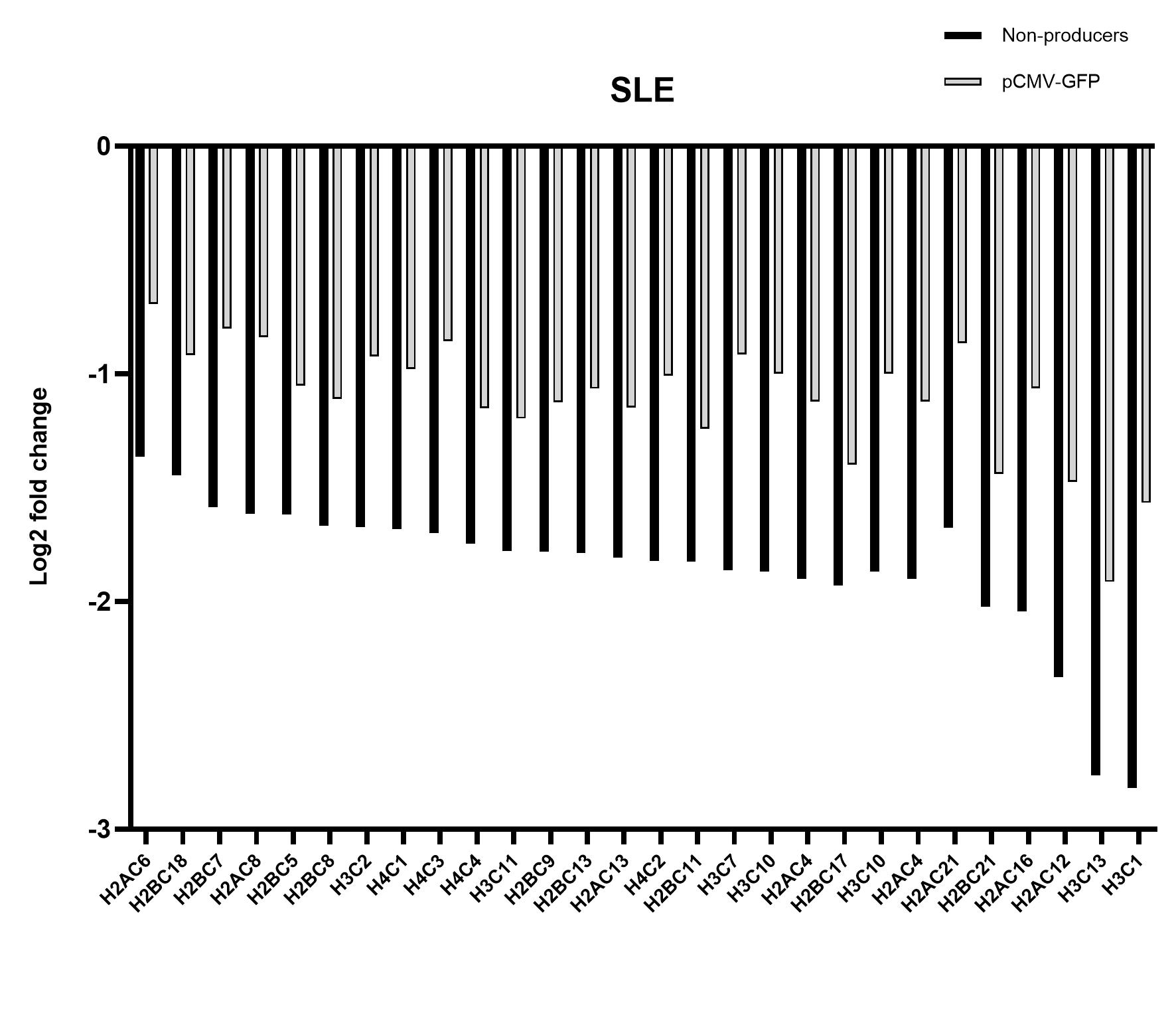


**Figure S4B Systemic Lupus Erythamatosus (SLE):** The relative levels of transcripts in the non-producer and pCMV-GFP samples compared to those in 293SF.
